# Agronomic and metabolomics analysis of rice-Tartary buckwheat (*Fagopyrum tataricum* Gaertn) bred by hybridization

**DOI:** 10.1038/s41598-022-16001-z

**Published:** 2022-07-14

**Authors:** Yan Wang, Zhixiu Guan, Chenggang Liang, Kai Liao, Dabing Xiang, Juan Huang, Chunyu Wei, Taoxiong Shi, Qingfu Chen

**Affiliations:** 1 Research Center of Buckwheat Industry Technology, Guizhou Normal University, Guiyang, 550001 China; 2 College of Bioengineering, Chengdu University, Chengdu, 610106 China; 3 National Research and Development Center for Coarse Cereal Processing, Chengdu, 610106 China

**Keywords:** Plant breeding, Metabolomics, Agricultural genetics

## Abstract

Tartary buckwheat (TB) is an edible pseudocereal with good health benefits, but its adhering thick shell and bitter taste inhibit its consumption. In this study, the first hybrid rice-Tartary buckwheat (RTB) variety Mikuqiao18 (M18), bred by the pedigree selection of crossbreeding ‘Miqiao’ (MQ) with ‘Jingqiaomai2’ (JQ2), was selected for an agronomic and metabolomics analysis. Compared with JQ2, M18 demonstrated a significantly lower yield per plant owing to the decreased grain weight and similar full-filling grain number per plant. However, M18 had a similar kernel weight per plant because of the thinner shell. The sense organ test suggested that M18 had higher taste quality regardless of partial replacement of rice through the improvement of preponderant indicators related to cereal taste quality, including lower values of total protein, albumin, glutelin, globulin, pasting temperature, cool paste viscosity, and setback. Meanwhile, M18 contained high levels of flavonoids, including rutin and quercetin, but presented a positive summary appraisal of cooking with 25% rice. Additionally, 92 metabolites were positively identified by GC–MS, including 59 differentially expressed metabolites (DEMs) between M18 and JQ2. Typically, M18 exhibited lower levels of 20 amino acids and higher levels of 6 sugars and 4 polyols. These DEMs might partly explain the superior eating quality of M18. In addition, M18 was abundant in 4-aminobutyric acid, which is beneficial to human health. The current findings offer a theoretical foundation for breeding rice-Tartary buckwheat with high yield and quality and promoting the cultivation and consumption of rice-Tartary buckwheat as a daily functional cereal.

## Introduction

Tartary buckwheat (TB, *Fagopyrum tataricum* Gaertn) and common buckwheat (*F. esculentum* Moench), native to southwest China and the Himalayas, are two dominant cultispecies of *Fagopyrum* Mill^1,2^. Common buckwheat is called ‘Tianqiao’ due to its good taste and has been suggested as a source of rutin in the daily diet for a long time^3^. Conventional Tartary buckwheat (CTB) is called ‘Kuqiao’ due to its bitter taste. Unlike common buckwheat, the shell of CTB is thick and adheres to the testa layer, contributing to increasing the difficulty of dehulling^1,4^. Traditional mechanical methods for the dehulling of CTB are complicated. Before dehulling, stewing and drying are two necessary steps, which not only raise the cost of processing but also lower the active ingredients of caryopsis^4,5^. Therefore, CTB is not as popular for daily consumption around the world despite containing several times more rutin than common buckwheat^6–10^.

‘Miqiao’ (MQ) is a special natural germplasm of TB and is also known as rice-Tartary buckwheat (RTB) given its thin shell for ready dehulling and cooking, similar to rice^11^. MQ is a local variety in Yunnan Province, China, with a long vegetative period and low yield characteristics. The poor agronomic traits of MQ were confirmed to be inherited when planted in many TB production areas^11,12^. In RTB breeding, genetic improvement for ecological adaptability and yield is recommended. Initially, Wang and Campbell reported the hybridization of RTB and found that the difficulty for crossbreeding was derived from the tiny flowers and low seed fertility^11^. Then, hot-water emasculation for TB was utilized in the crossbreeding of RTB^13^. Nonetheless, these hybrid RTB germplasms are rarely cultivated around the world due to defects such as poor ecological adaptability and low yield.

TB is classified as a cereal considering many similarities to other cereals. Starch and protein are the dominant storage components in CTB seeds. As the starch pasting viscosity and physicochemical properties of CTB are unique and different from most cereals, it has been suggested as an available source of retrograded starch^14^. Meanwhile, CTB seeds are abundant in proteins that are important active ingredients for reducing plasma total cholesterol^15^. CTB is famous for its high levels of flavonoids, which are recognized as the major active components for health care uses^16–18^. The cereal quality of RTB directly affects food development and processing. Compared with the CTB cultivar ‘Jinqiaomai2’ (JQ2), MQ exhibits similar levels of storage components, such as starch and proteins, while presenting significantly lower levels of flavonoids^12^. Considering that JQ2 is a high yield CTB cultivar with high levels of flavonoids, a series of RTB lines were created by crossing JQ2 with MQ^19,20^. Among them, Mikuqiao18 (M18) was identified and authorized as a high-yield variety. Therefore, the RTB variety M18 was selected to investigate the yield, major storage components, metabolites, starch pasting viscosity, and taste quality. The current findings will provide fresh insights into the ecological adaptability and cereal quality of RTB to popularize its planting, processing, and consumption as a daily food.

## Results

### Agronomic analysis

**Figure 1:**
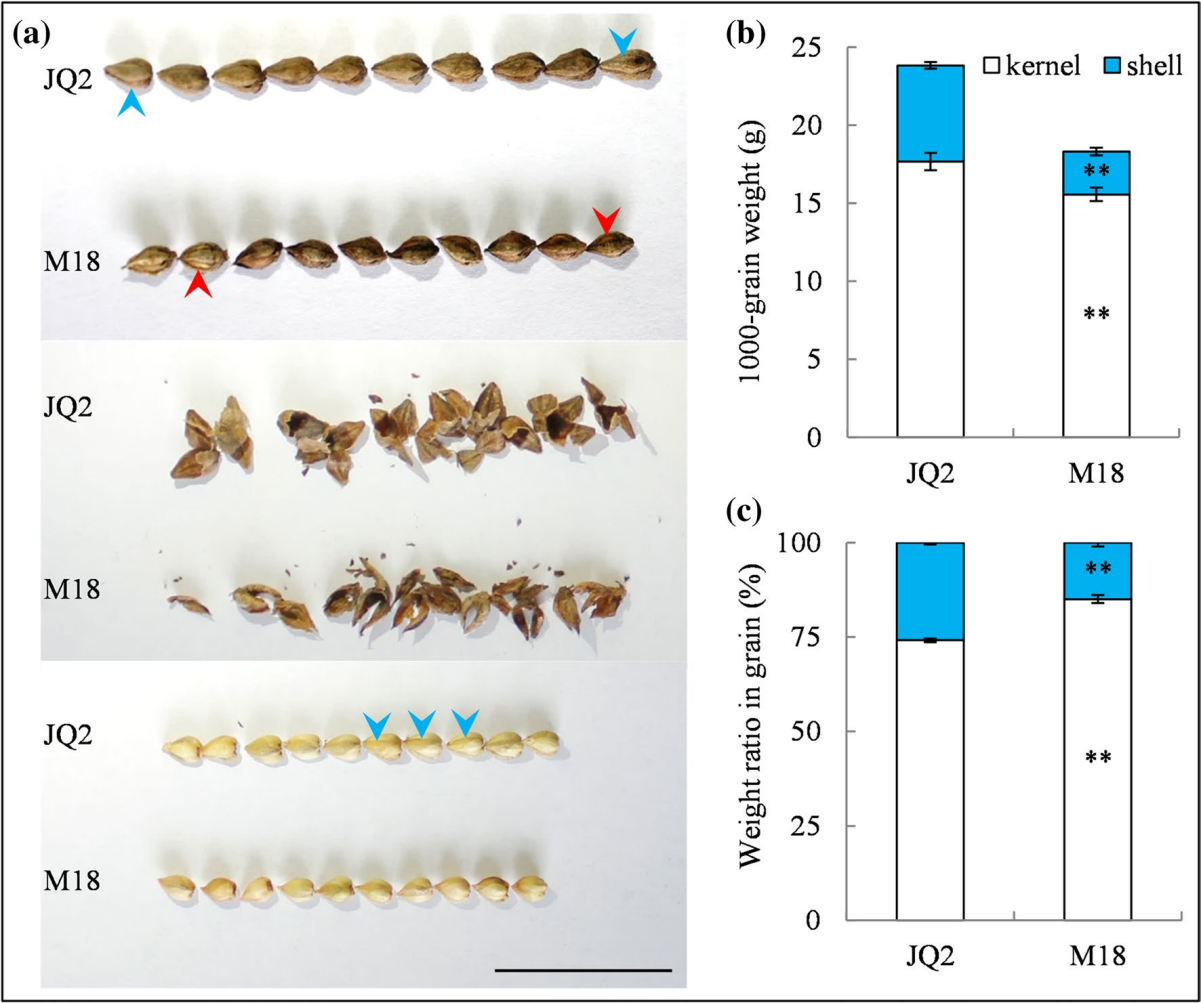
Seed phenotype (**a**), grain weight (**b**) and weight ratio of kernel and shell (**c**) in grains of Tartary buckwheat. The photo was spliced by PowerPoint in Microsoft Office 2003. Blue arrows point to the longitudinal furrows of CTB seed, and red arrows point to the cracked shell of RTB seed. JQ2 indicates Jinqiaomai 2, M18 indicates Mikuqiao 18. Bar = 2 cm. * and ** indicate significant differences at 0.05 and 0.01, respectively, according to independent samples *t*-test (n = 3).

An effective method for TB hybridization was reported, and a considerable number of hybridization combinations were performed in RTB breeding^19^. Afterwards, hundreds of RTB lines were planted for pedigree selection. Among them, an excellent line from crossbreeding JQ2 with MQ, designated Mikuqiao18 (M18), was identified and authorized as a high-yield RTB variety by 6 generations of pedigree selection. M18 typically demonstrated an easy dehulling property inherited from its mother parent MQ, and it displayed a visible shell phenotype differing from that of JQ2. Notably, the shell of M18 was thin and partly cracked without a longitudinal furrow that could not cover the kernel completely (Fig. 1a).

The RTB variety MQ exhibited a long vegetative period and low yield characteristics when planted in many production areas^11,12^. As M18 presented a longer period of growth, inherited from its mother parent, M18 was sown 10 days before JQ2 and achieved a similar maturing status in this experiment. At harvest, M18 exhibited extremely significantly higher plant height than JQ2 at harvest in two tested years^12^. Although the effective branching number was undifferentiated, the grain number per plant in M18 was extremely significantly increased. However, no significant difference in the full-filling grain number per plant between JQ2 and M18 was observed owing to the higher flat and empty grains in M18 (Table 1).

**Table 1:** Agronomic analysis of M18 planted in 2018 and 2019.

Year	Material	Plant height (cm)	Effective branching number	Grain number per plant	Full filling grain number per plant	Yield per plant (g)	Kernel yield per plant (g)	Yield (t/hm^2^)
2018	JQ2	110.7 B	7.1 a	324.8 B	197.1 b	4.6 a	3.4 a	1.8 a
	M18	130.1 A	7.2 a	476.1 A	219.6 ab	4.0 b	3.4 a	1.7 a
2019	JQ2	108.9 B	7.0 a	293.5 B	218.8 ab	4.9 a	3.5 a	1.9 a
	M18	132.2 A	7.6 a	488.9 A	236.9 a	4.4 b	3.5 a	1.9 a

Different uppercase and lowercase indicate the significant difference between M18 and the control variety at 0.05 and 0.01, respectively, according to one-way ANOVA.

Compared with JQ2, the 1000-grain weight of M18 was extremely significantly lower (Fig. 1b), while the kernel weight ratio in grain (85.0%) was extremely significantly higher, and the shell weight ratio in grain (15.0%) was extremely significantly lower (Fig. 1c). M18 obtained a parallel yield per plant, although the yield per plant was remarkably lower (Table 1).

### Rapid visco analyser detection and sense organ test

To identify the cooking and eating quality of RTB, the starch pasting viscosity parameters were detected using a rapid visco analyser (RVA). M18 demonstrated a significantly lower value of pasting temperature (PT) (61.0 °C) and an extremely significant lower cool paste viscosity (CPV) value, leading to significantly lower values of setback (SB) and consistency (CS) compared with JQ2 (Table 2). Nevertheless, no significant difference was detected in the peak viscosity (PV), hot paste viscosity (HPV), and breakdown (BD) between M18 and JQ2.

**Table 2:** The pasting viscosity parameters of Tartary buckwheat.

Material	PV (RVU)	HPV (RVU)	BD (RVU)	CPV (RVU)	SB (RVU)	CS (RVU)	PT (°C)
M18	125.6 a	122.1 a	3.5 a	214.3 B	88.7 b	92.2 b	61.0 b
JQ2	131.4 a	127.5 a	3.9 a	231.9 A	100.5 a	104.4 a	64.2 a

*PV* peak viscosity, *HPV* hot paste viscosity, *BD* breakdown, *CPV* cool paste viscosity, *CS* consistency, *PT* pasting temperature. Different uppercase and lowercase indicate the significant difference between M18 and the control variety at 0.05 and 0.01, respectively, according to independent samples *t*-test (n = 3).

Currently, people prefer to cook coarse cereals with rice together as a staple food to achieve nutritional and health benefits. Therefore, it might be acceptable to consume RTB kernel with rice daily. To further evaluate the taste of cooked kernel of RTB, the appearance, aroma, viscosity, and taste of different formulated M18 with rice were investigated according to the method of rice taste technology and compared with those of the famous rice variety ‘Koshihikari’. Consistently, both M18 and JQ2 exhibited lower values of appearance, aroma, viscosity, and taste when individually cooked (Table 3). Nevertheless, M18 exhibited higher values of appearance, taste, viscosity, and summary than JQ2. Moreover, the positive appraisal of the sense organ test was identified when TB kernel was mixed with rice. Note that the positive summary appraisal was tested when the additive amount of M18 reached 75%.

**Table 3:** Sense organ test of palatability characteristics on Tartary buckwheat.

Indicator	25% TB + 75% Rice		50% TB + 50% Rice		75% TB + 25% Rice		100% TB	
	M18	JQ2	M18	JQ2	M18	JQ2	M18	JQ2
Appearance	1.2	0.3	0.7	0.3	0.8	− 0.6	− 0.2	− 1.6
Aroma	1.0	0.7	0.8	− 0.1	0.2	− 0.6	− 0.9	− 0.8
Taste	1.3	0.4	0.7	− 0.3	− 0.3	− 1.5	− 0.2	− 1.8
Viscosity	1.0	0.8	0.9	− 0.2	0.1	− 0.8	− 0.4	− 1.1
Summary	1.2	0.7	0.8	0.1	0.2	− 1.1	− 0.8	− 1.7

*TB* Tartary buckwheat. Rice was set as the control.

### Storage component detection

**Figure 2:**
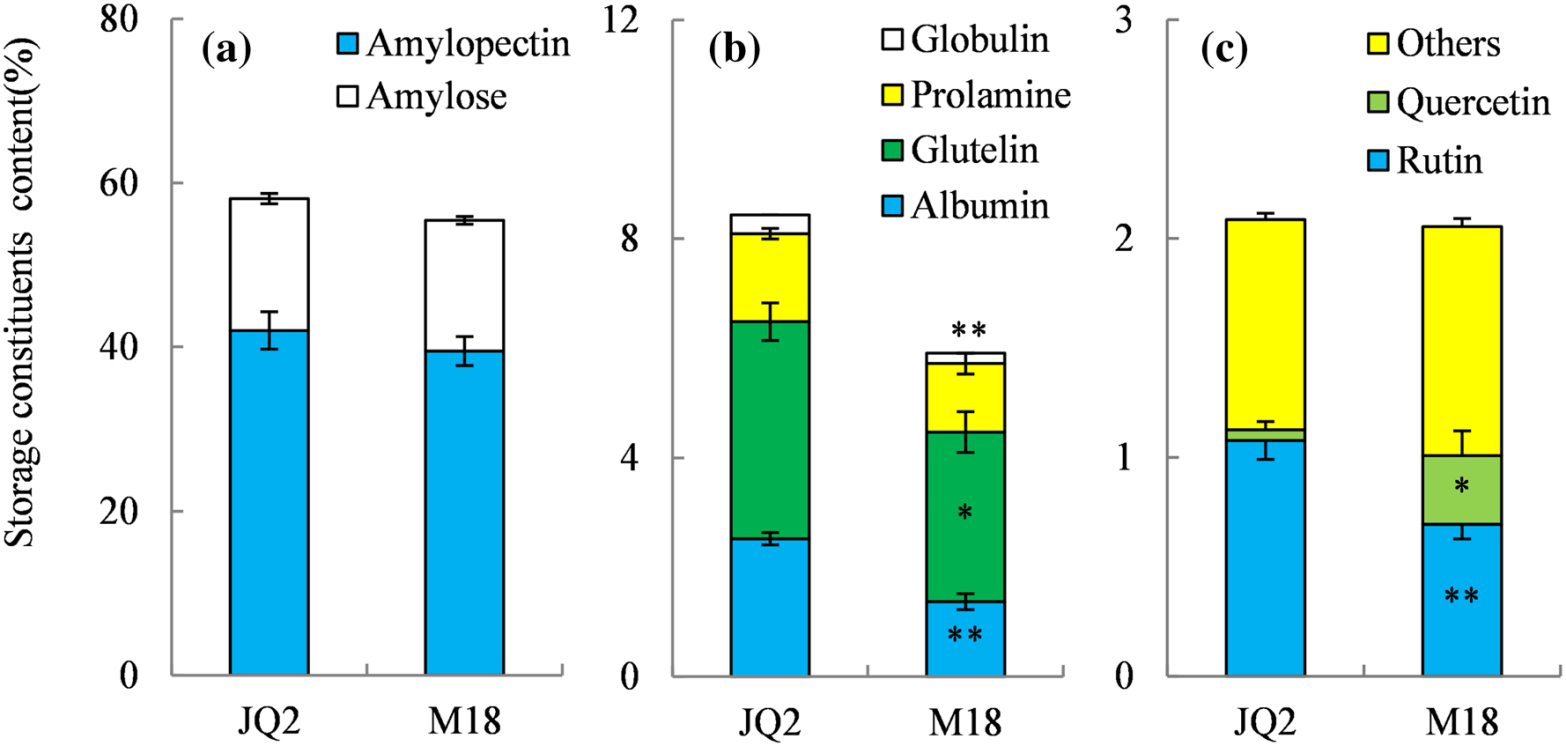
The levels of starch (**a**), proteins (**b**) and flavonoids (**c**) in grain of Tartary buckwheat. * and ** indicate significant differences at 0.05 and 0.01, respectively, according to independent samples *t*-test (n = 3).

As storage components are closely related to crop quality, the major storage components in kernels of M18 were determined. Starch was the dominant storage component in TB seeds (Fig. 2). Compared with JQ2, M18 demonstrated similar levels of starch consisting of amylose and amylopectin but significantly lower levels of total protein due to the significantly decreased albumin, glutelin, and globulin (Fig. 2b). Flavonoids are the dominant biologically active components in TB seeds, such as rutin and quercetin. Although no significant difference in total flavonoids was detected between M18 and JQ2, M18 presented extremely significantly lower contents of rutin and significantly higher contents of quercetin (Fig. 2c).

### Metabolomics analysis

**Figure 3:**
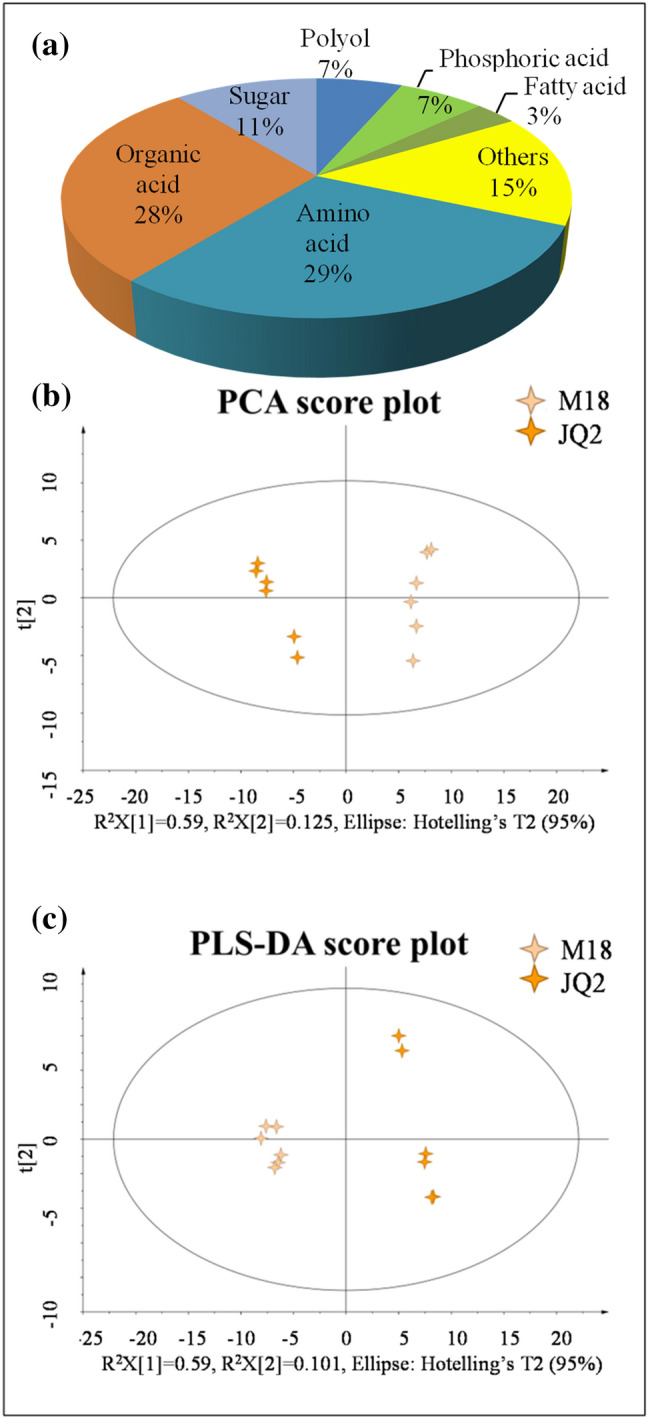
The groups (**a**), PCA (**b**) and PLS-DA (**c**) of metabolites in Tartary buckwheat grains by GC–MS. Multivariate statistical analysis was performed using the software package SIMCA-P (V13.0) and R language *ropls* package.

To identify the metabolites in M18, the GC–MS metabolomics platform was used. A total of 92 metabolites were positively identified, mainly including 27 amino acids, 26 organic acids, 10 sugars, 6 phosphoric acids, 6 polyols, and 3 fatty acids (Fig. 3a). The principal component analysis (PCA) indicated that the metabolomes were different among M18, JQ2, and the quality control, with R^2^X[1] and R^2^X[2] values of 0.59 and 0.125, respectively (Fig. 3b). OPLS-DA was performed to maximize the distinction between M18 and JQ2, with R^2^X[1] and R^2^X[2] values of 0.59 and 0.101, respectively (Fig. 3c), indicating that the established model was appropriate for differential expressed metabolites (DEMs) screening.

**Figure 4:**
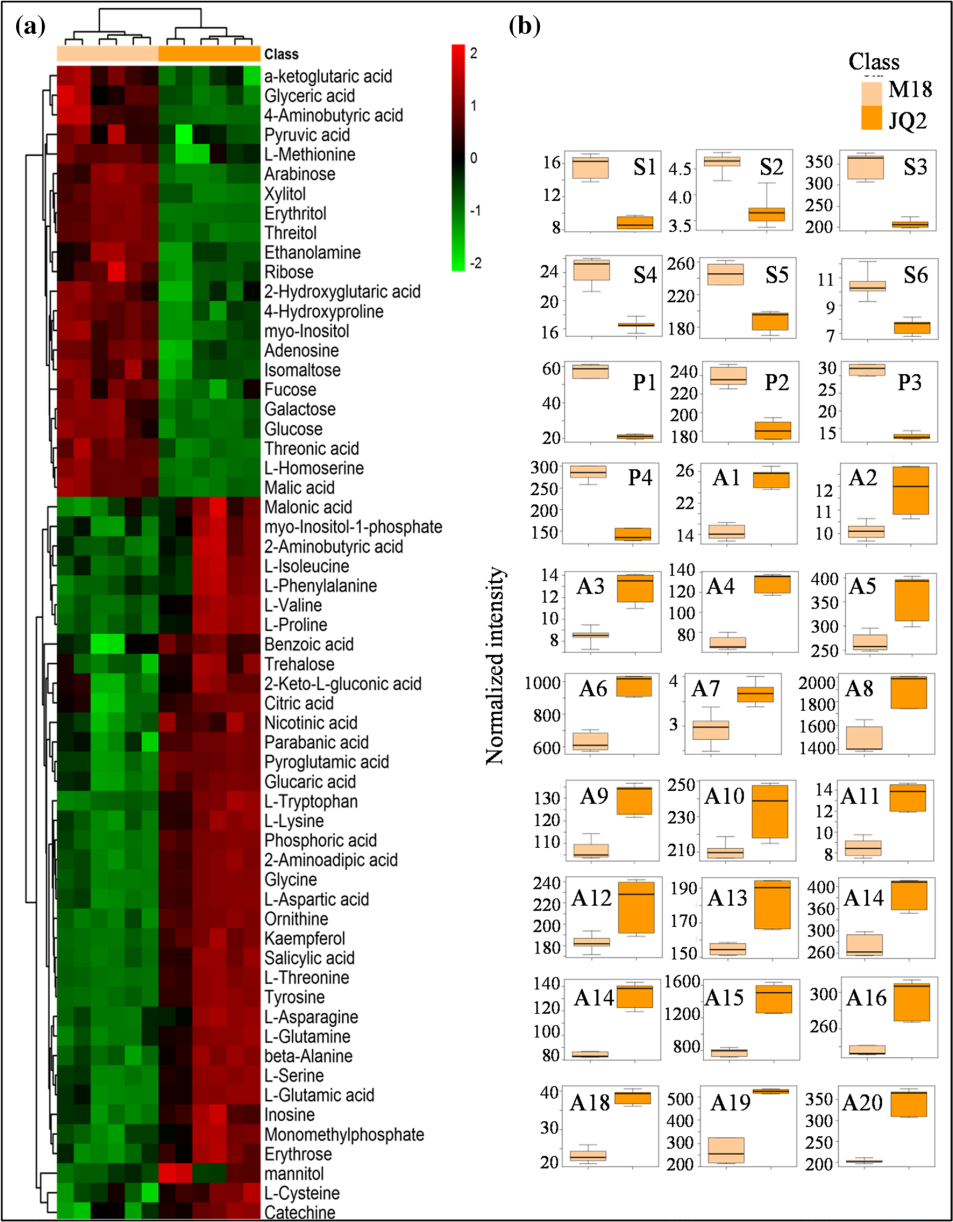
The heatmap (**a**) of DEMs and the boxplot-visualizations of partial DEMs (**b**) by GC–MS. The scale of data set and the bidirectional clustering of samples and metabolites by hierarchical clustering were performed using the pheatmap package in R (V1.0.8). S1 indicates arabinose, S2 indicates fucose, S3 indicates galactose, S4 indicates glucose, S5 indicates isomaltose, S6 indicates ribose, P1 indicates erythritol, P2 indicates myo-inositol, P3 indicates threitol, P4 indicates xylitol, A1 indicates 2-aminoadiopic acid, A2 indicates 2-aminobutyric acid, A3 indicates beta-Alanine, A4 indicates glycine, A5 indicates l-asparagine, A6 indicates l-aspartic acid, A7 indicates l-cysteine, A8 indicates l-glutamic acid, A9 indicates l-glutamine, A10 indicates l-isoleucine, A11 indicates l-lysine, A12 indicates l-phenylalanine, A13 indicates l-proline, A14 indicates l-serine, A15 indicates l-threonine, A16 indicates l-tryptophan, A17 indicates l-valine, A18 indicates ornithine, A19 indicates pyroglutamic acid, A20 indicates tyrosine. (n = 6).

The heatmap exhibited a great difference in DEMs between M18 and JQ2 (Fig. 4a). A total of 22 elevated and 37 reduced DEMs were identified in M18 (*p* value ≤ 0.05 and VIP value ≥ 1) compared with JQ2 (Fig. 4a, Table S1). Among them, M18 demonstrated elevated levels of 4-hydroxyproline (2.69-fold), l-homoserine (2.49-fold), and l-methionine (1.3-fold), but reduced levels of 20 amino acids, such as glycine (0.53-fold), l-tryptophan (0.53-fold), tyrosine (0.53-fold), and ornithine (0.53-fold), indicating that the metabolism of amino acids in M18 was weaker than that in JQ2 (Fig. 4b, Table S1). By contrast, M18 showed elevated levels of 6 sugars (arabinose (1.79-fold), fucose (1.25-fold), galactose (1.68-fold), glucose (1.47-fold), isomaltose (1.30-fold), and ribose (1.39-fold)) and 4 polyols (erythritol (2.73-fold), myo-inositol (1.31-fold), threitol (2.48-fold), and xylitol (2.02-fold)), implying that the metabolism of sugars and polyols was improved in M18 (Fig. 4b, Table S1). Meanwhile, M18 displayed elevated levels of metabolites associated with the tricarboxylic acid cycle, consisting of pyruvic acid (1.76-fold), a-ketoglutaric acid (1.62-fold), and malic acid (1.82-fold). Additionally, M18 showed a 2.3-fold increase in 4-aminobutyric acid compared with JQ2 (Table S1).

The KEGG analysis demonstrated that the DEMs covered 50 pathways or metabolisms. The most diverse metabolic pathways included aminoacyl-tRNA biosynthesis; arginine and proline metabolism; glycine, serine and threonine metabolism; and alanine, aspartate, and glutamate metabolism (Table S2).

**Figure 5:**
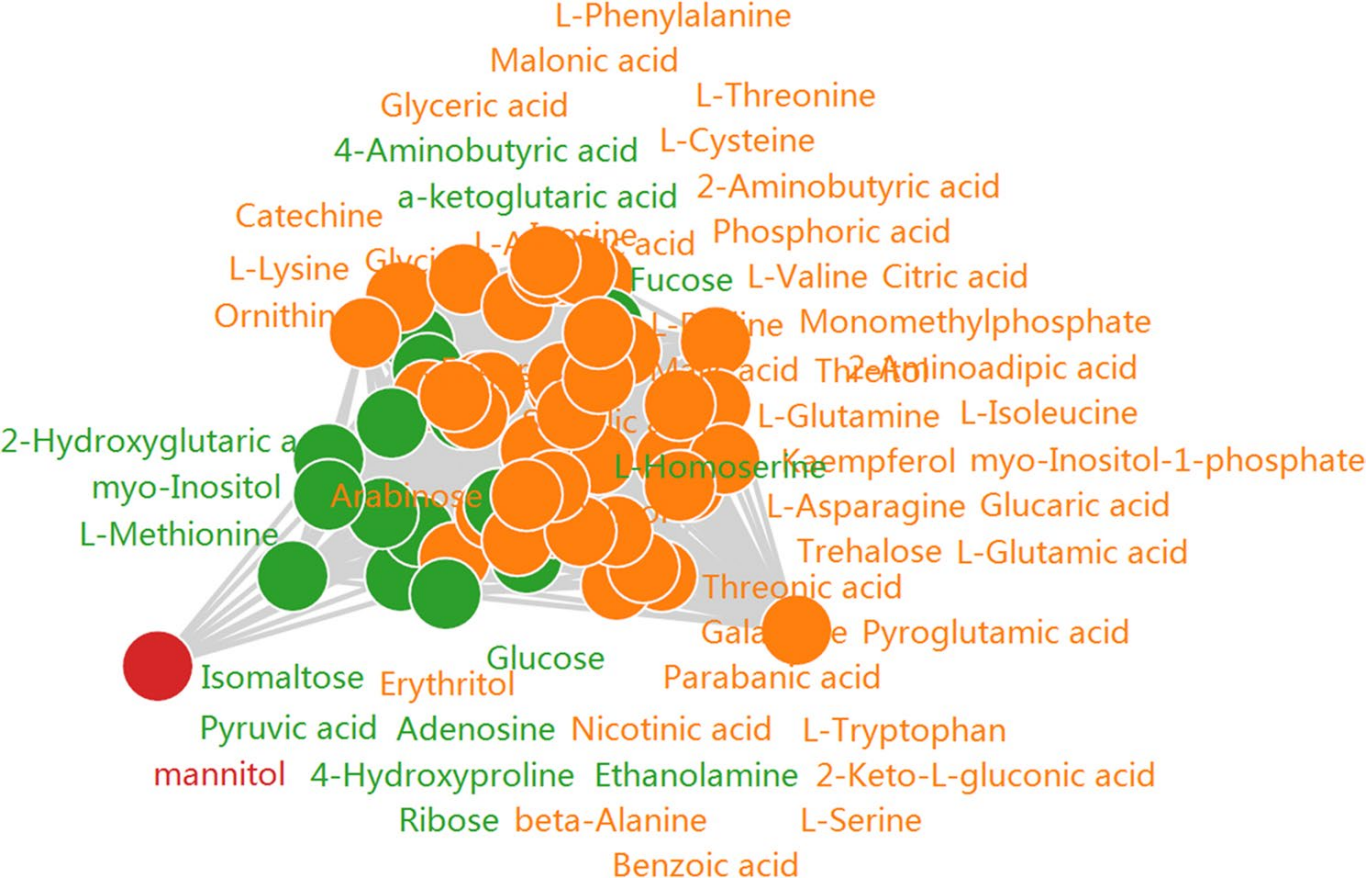
Pearson’s correlation coefficient analysis of DEMs by GC–MS. The correlation coefficient was calculated by Pearson correlation coefficient using the cor() function in R (V3.1.3). The significance statistical test of metabolite correlation analysis was conducted using the cor.test() function in R language package (|R| ≥ 0.7 and FDR *p* value ≤ 0.05).

Person’s correlation coefficient analysis revealed 1434 significant correlation coefficients (*p* < 0.01, *r*^2^ ≥ 0.49, FDR ≤ 0.01) between DEMs, including 822 positive and 612 negative significant correlations (Fig. 5, Table S3). Among them, l-homoserine, ornithine, erythritol, threitol, xylitol, and kaempferol were the most associated metabolites and all contained 57 cytonodes (Table S3). Negative significant correlations were observed between metabolites from different metabolic pathways, such as xylitol and phosphoric acid (− 0.993), xylitol and glycine (− 0.989), and xylitol and kaempferol (− 0.979). Meanwhile, positive significant correlations were discovered between metabolites belonging to the same metabolic pathways, such as l-aspartic acid and glycine (0.997), threitol and erythritol (0.995), and glucose and galactose (0.994) (Table S3).

## Discussion

### Crossbreeding of high-yield RTB for cultivation

Breeding adaptive RTB cultivars with easy dehulling seeds through hybridization using ‘Miqiao’ as a parent has long been considered. However, the crossbreeding of RTB is challenging due to the tiny flowers and low seed fertility^11^. Although the success of hot-water emasculation for RTB has been reported^13^, crossbreeding RTB is not popular with other research teams given some unknown mechanism that interferes with fertilization. Thus, RTB has not been widely planted around the world. Our previous work reported the success of hybridization of RTB by artificial emasculation on newly opened flowers using sharp materials such as toothpicks and tweezers^19^. Hereafter, hundreds of lines were created by crossing high yield CTB varieties with RTB germplasm MQ. Finally, M18, crossbred by MQ with JQ2, was identified and authorized as a high-yield RTB variety by 6 generations of pedigree selection. M18 exhibited an easy dehulling property inherited from its mother parent MQ^12^, and its shell was thin and accounted for only 15% of the grain weight. Additionally, M18 exhibited a significantly higher grain number per plant than JQ2, suggesting that the ‘sink’ was improved by crossbreeding. However, the full-filling grain number per plant of M18 was not significantly increased, and the grain weight was significantly decreased, leading to a significantly lower yield per plant compared with JQ2. Moreover, the kernel weight per plant of M18 was similar to that of JQ2 because of the significantly thinner shell. Although M18 showed a longer period of growth than JQ2, the poor yield characteristics of MQ were overcome in M18 as similar yield levels were achieved in two tested years compared with the high-yield variety JQ2^12^. Therefore, M18 was proposed as a potential RTB variety for cultivation in TB-producing areas.

### Crossbreeding of high taste quality RTB for daily consumption

Buckwheat is classified as a pseudocereal for its similar chemical composition as conventional cereals. CTB contains several times more flavonoids than common buckwheat and has long been suggested as a dietary source of rutin^21^. CTB is rarely directly cooked for daily consumption around the world because of its bitter taste, despite its excellent health benefits^22^. Therefore, the cooking and eating quality remains to be improved, even though the problem of dehulling is not present in RTB. Nonetheless, the taste quality of crops is a complicated trait controlled by multitudinous factors, such as seed storage components and metabolites.

Starch is the dominant storage component in CTB seeds and is composed of amylose and amylopectin. The pasting viscosity of cereal is dramatically influenced by the starch level, especially the amylose level. RVA has been widely employed to evaluate the starch pasting properties, cooking and taste quality of cereals^23–25^. In this study, the levels of starch, amylose, and amylopectin in M18 were similar to those in JQ2. Hence, no significant difference in the values of RV, HPV and BD between M18 and JQ2 was detected by RVA. In addition, the values of SB and CPV are significantly negative correlated with the cooking and taste quality of cereals^26^. Consistent results were revealed in TB, as higher values of appearance, viscosity, and taste and lower values of SB and CPV were discovered in M18 compared with JQ2.

Proteins are the major nutritional component in CTB seeds. High levels of proteins are proposed in high-quality breeding of cereals, while excessive levels of proteins negatively affect taste quality^27^. This relationship probably remained in TB considering that lower levels of most proteins (albumin, glutelin, prolamine) were detected in M18 while higher taste quality was found compared with JQ2. Therefore, the contradiction between nutritional quality and taste quality might exist and should be further improved in the crossbreeding of RTB for high yield and quality.

Flavonoids, especially rutin and quercetin, are the major active components in CTB seeds^16–18^. Compared with JQ2, M18 exhibited extremely significantly lower levels of rutin and significantly higher levels of quercetin. No significant difference in total flavonoid levels between JQ2 and M18 was detected, although the total rutin and quercetin in M18 was slightly lower than that of JQ2. Thus, the RTB variety M18 had a high capacity for health efficacy, similar to JQ2. High levels of rutin in CTB can be hydrolysed into quercetin during the cooking process and have been verified to be responsible for its bitter taste^21,28^. Furthermore, 87.5% of the rutin RTB flour degraded 10 min after the addition of water, while the rutin in RTB dehulled whole seed (whole groats) was stable even when it was immersed in water^21^. Currently, an increasing number of people prefer to eat rice mixed with coarse cereals daily to achieve health benefits. In this study, the sense organ test of palatability characteristics was investigated among different ratios of dehulled whole seeds of RTB mixed with rice. The positive appraisal of the sense organ test, including appearance, aroma, viscosity, and summary, was identified at the maximum level of 75% M18 mixed with 25% rice variety ‘cv. Koshihikari’. Therefore, it is feasible to directly utilize the dehulled whole seed of RTB cooked with rice.

### Metabolomics provides an effective strategy for RTB quality improvement

Metabolomics is a powerful strategy for determining metabolites and explaining macroscopic biological phenomena, providing new insight into crop improvement^29^. In this study, a total of 59 DEMs were identified between M18 and JQ2 by GC–MS. Amino acids are not only precursors for protein synthesis but also crucial nutritional components for cereals. Compared with JQ2, M18 had only three elevated amino acids (4-hydroxyproline, l-homoserine and l-methionine) belonging to the aspartic acid family. Most amino acids of the five families were reduced in M18, including the a-ketoglutaric acid family, pyruvic acid family, glycerate-3-phosphate family and aromatic amino acid family amino acids. Nonetheless, a-ketoglutaric acid and pyruvic acid were elevated in M18, suggesting that the synthesis of amino acids was limited by the deficiency of the nitrogen source instead of the carbon source. This was consistent with the results of the significantly lower protein contents in M18, which weakened the nutritional quality but might contribute to the better taste quality. M18 is a 4-aminobutyric acid-rich variety, the level of which is 2.3-fold that of JQ2, bringing many benefits such as improving sleep quality and lowering blood pressure^30^.

Tartary buckwheat is a well-known functional cereal called ‘kuqiao’ owing to its bitter taste^2,22^. Currently, the demand for high-quality Tartary buckwheat is gradually increasing in modern life. Sugars are not only energy sources but also important sweeteners in cereals. Compared with JQ2, M18 had 6 elevated sugars. Among them, arabinose was the most elevated sugar in M18, and it benefits the hypoglycemic effect by reducing disaccharide hydrolysis and lipid lowering by reducing the synthesis of fat^31^. Meanwhile, M18 showed elevated fucose, the conjugation of which plays an important role in cancer biology^32^. Polyols are well-known sugar-free sweeteners. Through GC–MS, 6 polyols were identified, while M18 presented 4 elevated polyols. Among them, the levels of erythritol, threitol, and xylitol were elevated more than twofold. As reported in rice, polyols vary in cultivars with different tastes^33^. Hence, these differentially accumulated metabolites might partly explain the superior eating quality of M18 by the sense organ test.

The cereal quality of Tartary buckwheat is a complex system consisting of nutritional quality, health care quality, and cooking and taste quality. It is difficult for traditional testing methods to comprehensively evaluate cereal quality^32^. In this study, the combined metabolomics and traditional testing methods provided an effective strategy for a comprehensive assessment of cereal quality, which would significantly accelerate the high-quality breeding process and promote the consumption of RTB as a daily functional cereal.

## Methods

### Plant materials and growth

The Tartary buckwheat cultivars ‘Jingqiaomai2’ (JQ2) and ‘Mikuqiao18’ (M18) were planted at the Experimental Station of the Research Center of Buckwheat Engineering Technology in Guizhou Province, China (1146.0 m, 26°50′ N, 106°58′ E) during the autumn growing season in 2018 and 2019. The average temperature was 21.79 °C/14.91 °C and 24.00 °C/16.55 °C, and the amount of precipitation was 329 mm and 244.2 mm during the growing period in 2018 and 2019, respectively (Supplement Table 2). JQ2 is a CTB high yield variety authorized by the National Identification Committee of Minor Grain and Bean in China. M18 is a high-yield RTB variety obtained by crossbreeding Miqiao with JQ2.

M18 was sown in early August, and JQ2 was sown in mid-August. However, they were synchronously harvested in late November. The soil in the test pot was yellow loam containing 12.15 g kg^−1^ organic matter, 0.81 g kg^−1^ total nitrogen, 96.37 mg kg^−1^ alkaline nitrogen, 12.42 mg kg^−1^ valid phosphorus, and 118.57 mg kg^−1^ valid potassium. The fertilizer included 60 kg N ha^−1^, 60 kg P_2_O_5_ ha^−1^, and 30 kg K_2_O ha^−1^, and was applied as a base fertilizer in each plot before sowing.

A randomized block design with three replications was used in each region. The area for each test plot was 2 m × 5 m, with row width of 33 cm. The planting density was approximately 1,000,000 plants per hectare. Consistent management measures were taken, and there were no significant pests, diseases, or weeds during the growth period.

### Plant sampling

A total of 12 separate plants from each plot in the Experiment Station of the Research Center of Buckwheat Engineering Technology were sampled at harvest to measure the plant height, effective branching number, grain number per plant, full-filling grain number per plant, grain weight, kernel weight, shell weight, yield per plant and kernel yield per plant. The rest of the plants in each plot were harvested to investigate the yield and quality.

### Measurement cooking and taste quality

A total of 3000 mg of sample and 25 mL of distilled water were added to a tube and detected by a rapid visco analyser (RVA, Newport Scientific, Australia). Parameters were determined according to Tone et al.^30^. Then, the values of PV, HPV, CPV, BD, SB, CS, and PT were analysed by software kit of Thermal Cycle Window according to Tone et al.^34^.

Regarding the identification of taste quality, M18 and JQ2 were mixed with rice variety ‘Koshihikari’ at 0, 25%, 50%, 75% and 100% ratios, respectively, and cooked in an electric rice cooker (MB-40EASY202, Midea, China). Twenty-two trained panelists participated in evaluating the taste quality from different aspects of appearance, aroma, taste, and viscosity, compared with Koshihikari and provided a summary appraisal following the method of Zhao et al.^35^.

### Measurement of storage components

TB kernels were previously crushed and sieved with a 100-mesh sieve for the further measurement of storage components.

A total of 100 mg of sample was eluted with 2 mL of 95% ethanol to remove soluble sugars three times. The details for amylose and amylopectin measurement were performed using the methods of Liang et al.^20^. The starch content was calculated with amylopectin and amylose.

A total of 100 mg of sample was used to extract albumin, glutelin, prolamine and globulin by adding 5 mL of 10 mmol/L Tris–HCl (pH 7.5), 5 mL of 0.5 mol/L NaCl, 5 mL of 60% normal propyl alcohol and 5 mL of mixed liquor (containing 0.5% sodium tartrate, 0.24% copper sulfate, 1.68% KOH and 50% normal propyl alcohol) three times. The albumin, glutelin, prolamine and globulin measurement were performed following the methods of Wang et al.^32^. The total protein content was calculated with the total albumin, glutelin, prolamine and globulin.

A total of 100 mg of sample was used to extract flavonoids by adding 2 mL of 80% ethanol to a hot bath at 70 °C for 5 h and ultrasonic extraction for 10 min. The flavonoids measurement was conducted following the methods of Wang et al.^36^.

A total of 100 mg sample was used to extract rutin by adding 20 mL methanol for ultrasonic extraction at 30 °C. The rutin measurement was achieved using the methods of Zhang et al.^37^.

A total of 600 mg of sample was used to extract quercetin by adding a moderate amount of ether 3 times for ultrasonic extraction at 30 °C for 30 min and 2 mL of ethyl acetate 5 times for ultrasonic extraction at 30 °C for 30 min to collect the supernatant. Then, the supernatant was dried at 80 °C and washed with sterile water. The sediment was collected by centrifugation and redissolved to a constant volume of 10 mL by ethanol. The quercetin measurement was performed following the methods of Lei et al.^38^.

### Measurement of metabolites

A total of 100 mg of fresh sample was ground by a high flux organization grinding apparatus under liquid nitrogen conditions. The sample preparation was conducted following the methods of Lisec et al.^39^ and Sangster et al.^40^. Metabolites were detected using GC (Agilent GC 7890A-5975C, USA) using a a 280 °C injection temperature, 1:20 split ratio, 150 °C interface, 230 °C ion source and temperature control (60 °C for 2 min, 10 °C/min rate up to 300 °C and holding there for 5 min). Then, mass spectrometry was recorded from 35 to 750 (m/z).

The data were processed by G1701 MSD ChemStation and R (v3.3.2) and analysed by AMDIS (v2.71) using NIST, Wiley Registry and GMD following the method of Smith et al.^41^. Multivariate data analysis was performed by SIMCA-P (v13.0, Umetrics, Sweden) and the R package *ropls*. The PCA and OPLS-DA were validated by the method of Thévenot et al.^42^. The potential biomarkers and variable contributions were evaluated through the analysis of VIP values (VIP > 1) and ANOVA (*p* value < 0.05) using SPSS (v19.0, IBM Corporation, Armonk, NY, USA). The heatmap was performed using the pheatmap package in R (V1.0.8, https://CRAN.R-project.org/package=pheatmap)^43^ KEGG analysis of DEMs was performed using the Kyoto Encyclopedia of Genes and Genomes database (http://www.kegg.jp/kegg/kegg1.html)^44,45^.

### Statistics and analysis

MS-Excel was used to classify Data. The means and differences of the data from three replications were determined by SPSS 17.0 (SPSS Inc., Chicago, IL, USA).

### Ethical statement

The permission of this study was obtained from Research Center of Buckwheat Engineering Technology in Guizhou Province. The experimental research and field studies on plants (either cultivated or wild) in this study were complied with relevant institutional, national, and international guidelines and legislation.

## Supplementary Information

Supplementary Table S1.

Supplementary Table S2.

Supplementary Table S3.

Supplementary Table S4.

## Data Availability

The datasets used and/or analysed during the current study available from the corresponding author on reasonable request.
